# *PNPLA3* Genotype, Arachidonic Acid Intake, and Unsaturated Fat Intake Influences Liver Fibrosis in Hispanic Youth with Obesity

**DOI:** 10.3390/nu13051621

**Published:** 2021-05-12

**Authors:** Roshonda B. Jones, Lide Arenaza, Claudia Rios, Jasmine F. Plows, Paige K. Berger, Tanya L. Alderete, Jennifer L. Fogel, Krishna Nayak, Passant Mohamed, Darryl Hwang, Suzanne Palmer, Frank Sinatra, Hooman Allayee, Rohit Kohli, Michael I. Goran

**Affiliations:** 1 Department of Pediatrics, The Saban Research Institute, Children’s Hospital Los Angeles, University of Southern California, Los Angeles, CA 90027, USA; rbarnerjones@gmail.com (R.B.J.); clrios@chla.usc.edu (C.R.); jplows@chla.usc (J.F.P.); paberger@chla.usc.edu (P.K.B.); jfogel@chla.usc.edu (J.L.F.); sinatra@med.usc.edu (F.S.); rokohli@chla.usc.edu (R.K.); 2 Institute for Innovation and Sustainable Development in Food Chain (IS-FOOD), Public University of Navarra, 31009 Pamplona, Spain; lide.arenaza@unavarra.es; 3 Department of Integrative Physiology, University of Colorado Boulder, Boulder, CO 80309, USA; Tanya.Alderete@colorado.edu; 4 Ming Hsieh Department of Electrical and Computer Engineering, University of Southern California, Los Angeles, CA 90007, USA; knayak@usc.edu; 5 Department of Radiology, University of Southern California, Los Angeles, CA 90033, USA; Passant.Mohamed@med.usc.edu (P.M.); darrylhw@usc.edu (D.H.); spalmer@usc.edu (S.P.); 6 Department of Preventive Medicine, University of Southern California, Los Angeles, CA 90033, USA; hallayee@usc.edu

**Keywords:** non-alcoholic fatty liver disease, liver stiffness, fibrosis, *PNPLA3*, genotype, arachidonic acid, unsaturated fat

## Abstract

Non-alcoholic fatty liver disease impacts 15.2% of Hispanic adolescents and can progress to a build-up of scared tissue called liver fibrosis. If diagnosed early, liver fibrosis may be reversible, so it is necessary to understand risk factors. The aims of this study in 59 Hispanic adolescents with obesity were to: (1) identify potential biological predictors of liver fibrosis and dietary components that influence liver fibrosis, and (2) determine if the association between dietary components and liver fibrosis differs by *PNPLA3* genotype, which is highly prevalent in Hispanic adolescents and associated with elevated liver fat. We examined liver fat and fibrosis, genotyped for *PNPLA3* gene, and assessed diet via 24-h diet recalls. The prevalence of increased fibrosis was 20.9% greater in males, whereas participants with the GG genotype showed 23.7% greater prevalence. Arachidonic acid was associated with liver fibrosis after accounting for sex, genotype, and liver fat (β = 0.072, *p* = 0.033). Intakes of several dietary types of unsaturated fat have different associations with liver fibrosis by *PNPLA3* genotype after accounting for sex, caloric intake, and liver fat. These included monounsaturated fat (β_CC/CG_ = −0.0007, β_GG_ = 0.03, *p*-value = 0.004), polyunsaturated fat (β_CC/CG_ = −0.01, β_GG_ = 0.02, *p*-value = 0.01), and omega-6 (β_CC/CG_ = −0.0102, β_GG_ = 0.028, *p*-value = 0.01). Results from this study suggest that reduction of arachidonic acid and polyunsaturated fatty acid intake might be important for the prevention of non-alcoholic fatty liver disease progression, especially among those with *PNPLA3* risk alleles.

## 1. Introduction

Non-alcoholic fatty liver disease (NAFLD) is the most common chronic liver disease in youths and is steadily increasing in line with childhood obesity [1]. Currently, the prevalence of NAFLD is 7.6% in the general pediatric population but increases to 34.2% in children and adolescents with obesity [2]. Data from NHANES has shown that NAFLD impacts 15.2% of Hispanic adolescents (compared to 10.1% of non-Hispanic Whites and 9.7% of non-Hispanic Blacks) [3]. NAFLD is a spectrum of disorders which begins with hepatic fat accumulation in the liver without excessive alcohol consumption [2] (steatosis) and can progress to non-alcoholic steatohepatitis (NASH), fibrosis, and eventually cirrhosis [4,5]. Although not all people with simple steatosis progress to the advanced disease, 30–40% of people with NAFLD develop NASH, with almost half of those patients progressing to liver fibrosis. Liver fibrosis is characterized by the accumulation of extracellular matrix proteins due to repeated injuries to the liver tissue and is the strongest predictor for disease-specific mortality for NAFLD [6].

NAFLD have been extensively studied in the last few decades. Studies have revealed that obesity, particularly abdominal obesity, as well as lifestyle habits, such as high carbohydrate and added sugar intake, and low levels of physical activity, contribute to NAFLD [7]. Conversely, the consumption of non-starchy vegetables has been shown to be protective against liver fat deposition in Latino youth [8]. Additionally, sex, age, and genetics may also be related to the likelihood of developing NAFLD, with older males having around 16% higher prevalence [9]. Specifically, individuals with a C to G polymorphism in their patatin-like phospholipase 3 (*PNPLA3*) gene are more susceptible to increased liver fat [10]. This *PNPLA3* polymorphism is prevalent in 50% of the Hispanic population, revealing an ethnic predisposition for hepatic steatosis [11,12].

The pathogenesis of liver fibrosis involves an inflammatory response, and dietary components have been shown to help modulate the inflammatory processes [9]. The Western diet, which is rich in omega-6 and low in omega-3 fatty acids, has been associated with a proinflammatory state [13,14]. High cholesterol intake might also promote liver fibrosis [15]. Liver fibrosis, while difficult to treat, may be reversible if diagnosed early [16,17]; therefore, it is of high interest to identify possibly modifiable risk factors contributing to its development. Despite this, little is known about predictors of liver fibrosis and biological or lifestyle factors that might trigger the progression of the disease from steatosis to fibrosis. Our previous research has determined differential associations between carbohydrate intake and liver fat by *PNPLA3* genotype [18]. Therefore, with a specific focus on Hispanic adolescents with obesity, this current cross-sectional study aims to: (1) identify potential biological predictors of liver fibrosis (i.e., liver fat, sex, *PNPLA3* polymorphisms, and diet); and (2) determine if associations between dietary components and liver fibrosis differs by *PNPLA3* genotype.

## 2. Materials and Methods

### 2.1. Study Design and Participants

Fifty-nine adolescents aged 11–18 years were included for the current cross-sectional analysis. Participants were recruited for two different studies at the University of Southern California which required identical baseline measurements including the collection of 24-h dietary recalls and clinical assessments. Data collected included baseline measures from 42 Hispanic adolescents (12–18 years of age) with obesity who were recruited from the ongoing HEROES (Healthy Eating through Reduction Of Excess Sugar), a 12-week intervention aimed to improve liver health through the reduction of sugar (clinical trial registered at www.clinicaltrials.gov: NCT02948647). Baseline measures of 17 Hispanic adolescents (12–19 years of age) with obesity were from a 16-week parallel, double-blind, and placebo-controlled trial examining the efficacy of probiotic supplementation in altering gut microbiota and gut hormones (clinical trial registered at www.clinicaltrials.gov: NCT03115385) [19] were included. The inclusion criteria for the current analysis including: (1) presence of obesity defined by a BMI percentile ≥95th for age and sex and (2) self-identified Hispanic ethnicity based on if the study subject, both parents, and all four grandparents have their origins in Cuba, the Dominican Republic, Mexico, Puerto Rico, South or Central America. Participants were excluded if they had diabetes or other diagnosed metabolic diseases, were participating in a weight-loss exercise program, were taking any prescription medication, were smokers, or were pregnant. Written parental consent and child assent for inclusion in these studies were obtained prior to any testing procedure for participants under 18 years of age. Studies were approved by the University of Southern California Institutional Review Board and were conducted in accordance with the Declaration of Helsinki.

### 2.2. Anthropometry, Adiposity, and Liver Fat and Fibrosis

Height, weight, and waist circumference were measured following standard protocols. Body fat and lean mass were measured by Dual-energy X-ray absorptiometry (DEXA) using a HOLOGIC QDR 5400 densitometer (Hologic, Inc., Bedford, MA, USA). Abdominal fat distribution (visceral fat versus subcutaneous) and liver fat was assessed by MRI using 3-Tesla MRI scan (Excite HD; GE Healthcare, Waukesha, WI, USA) [19]. The previously validated IDEAL method was used during the MRI scans, which consisted of separating water and fat components divided by the sum of the fat and water components using chemical-shift MRI [19]. Liver fibrosis was measured by Magnetic Resonance Elastography (MRE), a non-invasive technology for measuring tissue stiffness that can determine the presence and stage of liver fibrosis by analyzing the propagation of shear waves transmitted into the abdomen [20]. This magnetic resonance imaging technique has been validated against liver fibrosis determined by liver biopsy. We used the pediatric population threshold previously proposed by Schwimmer et al., which considers adolescents with MRE values of <2.74 kPa to have no fibrosis; while MRE values of ≥2.74 kPa have stage 1 or greater fibrosis [20].

### 2.3. Genotype

Blood samples were collected from participants and genomic DNA was isolated for genotype analysis. Genotyping for SNP rs738409 in *PNPLA3* was performed using the Applied Biosystems, Inc., Foster City, CA, USA, (ABI) TaqMan system.

### 2.4. Dietary Intake

A trained registered dietitian conducted two 24-h dietary recalls at each time point using the Nutrition Data System for Research (NDSR) software (version 2018) with the multiple pass technique. The first recall was performed in person at our laboratory with the use of food models, portion booklets, or serving containers to assist in estimating serving sizes. The remaining recalls were conducted by telephone. To minimize the potential for undereating or underreporting in the time frame for subsequent recalls, participants were not aware of the telephone recall schedule.

### 2.5. Statistical Analysis

To test for normality, we used Shapiro–Wilk’s test and variables whose distributions were significantly different from the normal distribution were log-transformed. Independent T-tests (continuous variables) and chi-square tests (categorical variables) were carried out to examine differences according to *PNPLA3* genotype. Genotype data was tested for deviations from Hardy–Weinberg equilibrium to ensure that the allele frequencies are as expected relative to the population.

We first examined the univariate associations between liver stiffness and biological characteristics using linear regression analysis for continuous variables and one-way ANOVA models for categorical models. Using multivariable linear models, we subsequently adjusted for *PNPLA3* genotype which is known to influence liver health [21] and also adjusted for the residuals of a simple model regressing liver fibrosis over liver fat. Next, we determined the associations between individual dietary components and liver stiffness using linear regression models with sex, caloric intake, and residuals of a simple model regressing liver fibrosis over liver fat included as covariates. As a second aim, we examined differential associations between dietary components and liver fibrosis by *PNPLA3* genotype. If we determined a significant association between a particular dietary component and liver stiffness, the association was further examined by determining the main food sources of the dietary component and examining whether this main food source was associated with liver stiffness. All of the analyses were carried out using SPSS statistical software version 20.0 (SPSS Inc.) and the R statistical programming language version 3.6.1.

## 3. Results

### 3.1. Description of Cohort

Descriptive biological characteristics of the 59 Hispanic adolescent participants (average age 14.2 ± 2 years, 50.8% females) are shown according to genotype (CC, CG, or GG) in Table 1. Participant genotyping determined that 30% of females and 44.8% of males possessed the GG genotype in the *PNPLA3* gene. Genotype frequencies were consistent with Hardy–Weinberg equilibrium (chi-squared test *p* = 0.20) with *p* = 0.58 minor allele frequency (G allele). Among the participants, 64.4% (n = 38/59) had NAFLD (as determined by a liver fat percentage ≥5.5%) and 16.9% (n = 10/59) of participants had liver fibrosis (as determined by an MRE value ≥ 2.74 kPa). Of the 10 participants with liver fibrosis, 8 were male and 2 were female (chi-square *p* = 0.03); additionally, 1 participant had the CC genotype, 2 had the CG genotype, and 7 had the GG genotype in the *PNPLA3* gene (chi-square *p* = 0.06). Of the biological characteristics examined, liver fat (*p* < 0.001) and stiffness (*p* < 0.05) were significantly higher in participants with the GG genotype compared to those with the CG or CC genotype. We found that liver fat in participants with the GG genotype was 2.03 and 2.63 times higher than in those participants with GC and CC, respectively. Liver fibrosis was 2.0 times higher in participants with the GG genotype compared to GC genotype and 7.0 times higher compared to CC genotype. Body composition measurement, macronutrients intake, and dietary fatty acid profile were all similar among genotype groups. None of the 59 participants in this study consumed supplements so nutrients examined are sourced from food.

### 3.2. Associations between Biological Characteristics and Liver Fibrosis

As shown in Table 2, genotype (mean CC/GC = 2.3 kPa, mean GG = 2.7 kPa, *p*-value = 0.003), sex (mean males = 2.6 kPa, mean females = 2.3 kPa, *p*-value = 0.002), and liver fat (β = 0.33, *p*-value = 0.01) were all associated with liver fibrosis. Both males and participants with GG genotype represent approximately 14% higher values of liver fibrosis. In addition, sex and genotype were significantly associated with liver fibrosis independent of liver fat and age but not when accounting for the influence of the interaction between sex and genotype on liver fibrosis (Supplemental Table S1).

### 3.3. Associations between Dietary Intake and Liver Fibrosis

**Figure 1:**
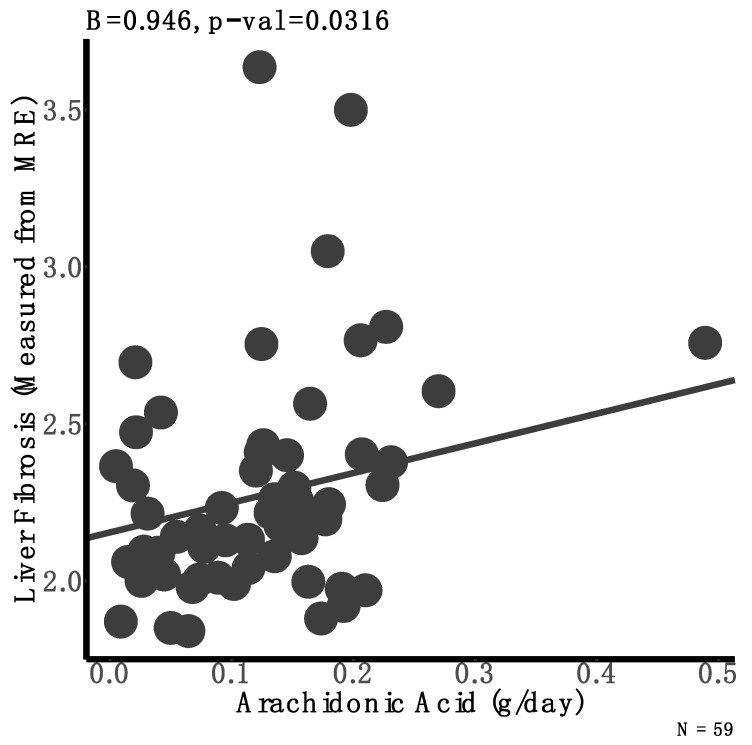
Association between arachidonic acid intake and liver fibrosis taking into account gender and genotype. A linear model was used to determine the association between arachidonic acid intake (g/day) and liver fibrosis. These models adjusted for sex, genotype, and residuals of a simple linear model regressing liver fat against liver fibrosis.

We next aimed to determine the influence of dietary intake on liver fibrosis, adjusting for sex, genotype, and liver fat. We found that arachidonic acid was positively associated with liver fibrosis (β = 1.14, *p* = 0.03) (Figure 1, Supplemental Table S2) such that the intake of every 1 g of arachidonic acid was associated with 37.7% higher liver fibrosis. Although the difference was not statistically significant, adolescents with liver fibrosis consumed 58% more arachidonic acid in their diet compared to their peers with no fibrosis (0.19 vs. 0.12 g of arachidonic acid intake for participants with and without liver fibrosis, respectively; *p* = 0.15). We found that the main dietary sources of this omega-6 fatty acid among our study sample was meat. This was confirmed with the positive association between arachidonic acid intake and meat consumption (Supplemental Figure S1).

### 3.4. Differential Associations between Dietary Intake and Liver Fibrosis by Genotype

**Figure 2:**
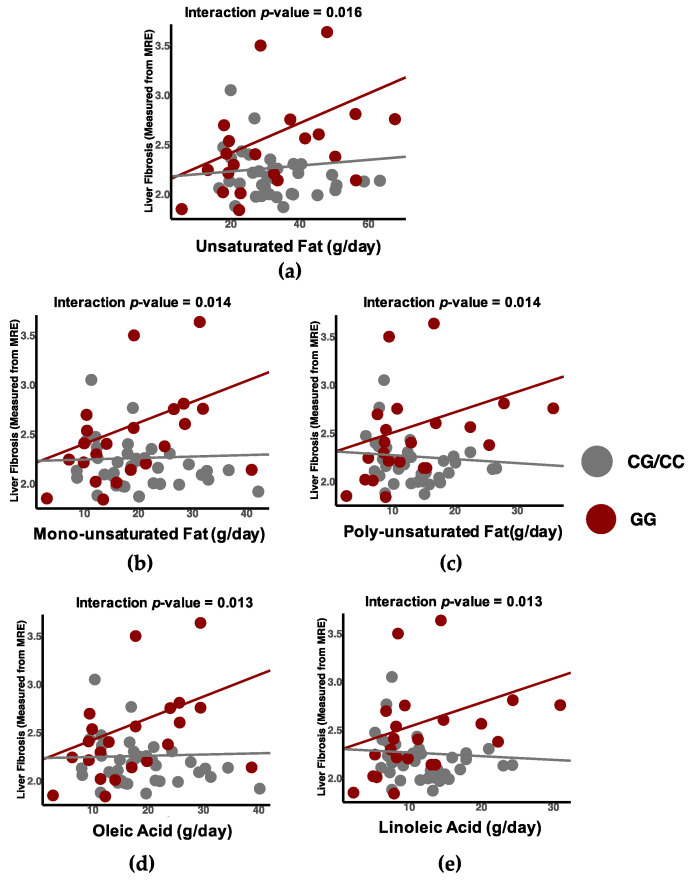
Dietary components with differential associations with liver fibrosis by *PNPLA3* genotype. Association between intakes of (**a**) unsaturated fat, (**b**) monounsaturated fat, (**c**) polyunsaturated fat, (**d**) oleic acid, and (**e**) linoleic acid and liver fibrosis differed by *PNPLA3* genotype. In participants with two copies of the *PNPLA3* gene 1148M variant allele (GG genotype), the association between liver fibrosis and unsaturated fat intake trends in a positive direction.

Finally, we examined differential associations between macronutrient intake and liver fibrosis by genotype independent of liver fat and sex. We found that participants with the GG genotype of the *PNPLA3* gene exhibited a positive association with total fat intake (β_CC/CG_ = 0.0002, β_GG_ = 0.00678, *p*-value_interaction_ = 0.02), monounsaturated fats (β_CC/CG_ = 0.002, β_GG_ = 0.02, *p*-value_interaction_ = 0.01), polyunsaturated fats (β_CC/CG_ = −0.004, β_GG_ = 0.02, *p*-value_interaction_ = 0.01), oleic acid intake (β_CC/CG_ = 0.001, β_GG_ = 0.02, *p*-value_interaction_ = 0.01) and linoleic acid intake (β_CC/CG_ = −0.004, β_GG_ = 0.02, *p*-value_interaction_ = 0.013) (Figure 2, Supplemental Table S2). The differential association of fats and liver fibrosis by genotype appeared to be driven by linoleic acid and oleic acids, which we found the main source to be from plant-based oils, sauces, and condiments as shown in Supplemental Figure S2.

## 4. Discussion

The current study examined the biological and dietary predictors of liver fibrosis in Hispanic youth with obesity and highlights several important findings. First, both sex and *PNPLA3* genotype were shown to contribute to greater liver stiffness independent of liver fat. Males and participants with the GG variant of the *PNPLA3* gene had higher values of liver fibrosis. Second, the dietary intake of arachidonic acid was significantly associated with liver fibrosis, suggesting that arachidonic acid is a dietary predictor of liver fibrosis. Lastly, we found that certain dietary fats had a direct association with liver fibrosis in the participants with the GG genotype but not the CC/GC genotypes, and this association was independent of sex, caloric intake, and liver fat.

More than a third of the study participants had the GG variant of the *PNPLA3* gene, which is similar to previously reported studies in the Hispanic pediatric population [18,22]. It is known that the prevalence of this genetic variation is higher in the Hispanic population compared to other ethnic/racial populations [10]. In accordance with the scientific literature [10,18,22,23,24], liver fat in participants with the GG genotype was 2.03 and 2.63 times higher than in those participants with GC and CC, respectively, which in turn is related to the increased prevalence of NAFLD in the Hispanic population. Interestingly, liver stiffness and the prevalence of liver fibrosis were also significantly higher in participants with the GG *PNPLA3* allele. Similarly, Valenti et al. [25] reported that homozygosity for *PNPLA3* influenced liver fibrosis in adults with NAFLD, indicating that this genotype is not only associated with the risk of developing NAFLD, but also with its progression to necroinflammation and fibrosis. As such, these findings support the evidence that there might be a genetic susceptibility for NAFLD development and progression to fibrosis which is more readily observed in the Hispanic population with the *PNPLA3* genotype, and that this process begins early in life [12].

Liver fat, sex, and *PNPLA3* genotype were all shown to be biological predictors of liver fibrosis. In addition, both sex and genotype were predictors of liver fibrosis independent of liver fat. Specifically, male sex was associated with a higher likelihood of developing liver fibrosis compared to females who presented with lower values and prevalence of liver stiffness and fibrosis, respectively. This is in line with other studies not only showing a higher prevalence of hepatic steatosis in males [26], but also that men display a higher risk of advanced fibrosis compared to premenopausal women [27]. This finding may be due to the protective-effects of estrogen in females during the child-bearing years, similar to what has been shown to occur with cardiovascular disease [27]. Furthermore, and as expected, participants with liver fibrosis presented with hepatic steatosis since all of them had increased liver fat deposition above 5.5% [28].

In addition to the identified biological predictors, we found that liver fibrosis may also be influenced by diet. One of these was arachidonic acid, a 20-carbon omega-6 polyunsaturated fatty acid (20:4) involved in inflammatory pathways and present in animal-origin foods. While arachidonic acid has been shown to contribute adversely to cardiovascular health due to inflammation, the exact mechanism is not completely understood [29,30]. Arachidonic acid not only is involved in several physiological mechanisms, but it is also the precursor for lipid mediators known as eicosanoids. Although this fatty acid can have anti-inflammatory and pro-resolving effects, arachidonic acid is predominantly pro-inflammatory [31]. Several aggregatory mediators of arachidonic acid, Prostaglandin E_2_ (PGE_2_), thromboxane A_2_ (TXA_2_), and B_4_ leukotriene (LTB_4_), are also known to be inflammatory since they are involved in processes such as vascular permeability and vasodilatation as well as platelets activation and aggregation [13]. A diet rich in omega-6 fatty acids may cause an accumulation of arachidonic acid in the cell membrane [32] due to its ability to alter the activity of delta 5- and 6-desaturase, resulting in increased arachidonic acid production and its derived eicosanoids while lowering that of eicosapentaenoic fatty acid (EPA) [33]. Moreover, decreased activity of delta 5- and 6-desaturase has been shown in the liver of obese patients with NAFLD [34].

In line with this, there is evidence that a diet rich in arachidonic acid inhibits the anti-inflammatory effects of the omega-3 fatty acids, EPA, and docosahexaenoic fatty acid (DHA) [31,35]. A lower EPA/arachidonic acid ratio (<0.21) has also been associated with fibrosis tissue [36]. Some studies suggest that an increased ratio of omega-3 to omega-6 fatty acids, and its effects of increasing arachidonic acid-derived eicosanoids should be taken into consideration in the pathogenesis of NAFLD [37,38]. In agreement with this hypothesis, we observed a trend to a lower EPA/arachidonic acid ratio in our study participants who had fibrosis. Since arachidonic acid and EPA are competitors for incorporation into the membrane phospholipids, EPA supplementation has been suggested as prevention of NAFLD progression. Although some studies suggest that omega-3 supplementation may help decrease liver fat [39,40], the evidence is not strong enough to recommend it as a treatment [41]. Nonetheless, Scorletti et al. reported improvements in liver fat with DHA and EPA treatment with no beneficial effects in fibrosis score [42]. Additionally, we examined our participants’ diet to determine that their main dietary source of arachidonic acid came from meat. This was confirmed by a positive association between meat consumption and arachidonic acid intake. Furthermore, we observed that chicken and poultry were the main meat contributors of arachidonic acid in their diets, possibly due to their habitual consumption of chicken nuggets.

Lastly, we found that overall fat intake had a differential association with liver stiffness by *PNPLA3* genotype. The *PNPLA3* gene encodes a protein that is structurally similar to the enzyme that is involved in triglyceride hydrolysis, adipose triglyceride lipase (*ATGL/PNPLA2*) [43]. The I148M substitution leading to the *PNPLA3* polymorphism may prevent catalytic activity by impacting the hydrophobic substrate binding site resulting in increased triglyceride content in the liver [44]. Thus, those with the *PNPLA3* variant can be prone to increased sensitivity to hepatic stress due to excess calories from diet [45]. As our previous research has determined differential associations between carbohydrate intake and liver fat by *PNPLA3* genotype [18], we were particularly interested in how the *PNPLA3* genotype impacts the association between diet and liver fibrosis in this current cohort.

The current study found that intake of unsaturated fat from food was differentially associated with liver stiffness by *PNPLA3* genotype, independently of liver fat, and that this finding was likely driven by the fatty acids, linoleic acid and oleic acid. It has been shown that the presence of linoleic acid and oleic acid is associated with increased expression of *PNPLA3* [46] and overexpression of *PNPLA3* in those with I148M variant has been shown to lead to steatosis [44,47]. Linoleic acid is an 18-carbon omega-6 polyunsaturated fatty acid (18:2) that is one of the two essential fatty acids. In mice, linoleic acid was essential in the development of alcoholic liver damage [48] and while the fibrosis in our cohort is not related to alcohol it could be reasoned that the overconsumption of linoleic acid impacts liver fibrosis. Work by Jeyapal et al. has provided evidence that replacing dietary linoleic acid with alpha-linoleic acid can inhibit diet-induced NASH in rats fed a high-fat, high-fructose diet [49]. Oleic acid is an 18-carbon monounsaturated omega 9 fatty acid (18:1n-9) and it is widely known to induce hepatic steatosis in the human hepatocyte-derived cell line HepG2 [50,51]. Cansanção et al. found that in a group of older adults, the percentage of oleic acid in red blood cells was higher in adults with advanced liver fibrosis although overall percent of monounsaturated fatty acids (MUFA) in red blood cells was not highly correlated with dietary intake of MUFA [52]. While these studies have shown associations between oleic acid and steatosis, how intake of dietary oleic acid can lead to liver fibrosis independently of liver fat remains unknown.

The current study is strengthened by the use of MRE to measure liver fibrosis, participants that possess similar characteristics according to ethnicity and obesity status, as well as dietary intake collection and analysis. However, given that the design of the study was cross-sectional and represents only one time point, we must stress that the findings in the current study are merely associations. In order to determine causality, a randomized-controlled trial is necessary. Nevertheless, the current research provides a sound basis for future work to determine the causal factors of liver fibrosis in this cohort.

## 5. Conclusions

In conclusion, the findings of the current study suggest that sex, *PNPLA3* genotype and liver fat might be biological predictors of liver fibrosis while arachidonic acid intake, predominantly obtained from meat consumption, appears to be a dietary predictor of liver fibrosis in Hispanic youth with obesity. Furthermore, we found that unsaturated fat was associated with liver stiffness differentially by *PNPLA3* genotype. Hence, the consumption of specific unsaturated fatty acid intake proportion may be of interest to prevent NAFLD progression; however, further research is needed to design specific guidelines.

## Figures and Tables

**Table 1:** Biological characteristics and dietary intake of participants in the study according to *PNPLA3* genotype.

	N	CC (n = 13)	N	CG (n = 24)	GG (n = 22)	*p*-Value
Biological Characteristics						
Age (years)	13	14.2 (2)	24	14.4 (2)	14.1 (2)	0.85
Females (N (%))	13	8 (61.5)	24	13 (54.2)	9 (40.9)	0.46
BMI (kg/m^2^)	13	35.7 (8.8)	24	33.7 (5)	32.8 (5.6)	0.19
Waist circumference (cm)	13	107 (16)	24	104 (12)	103 (11)	0.43
Body fat (%)	13	42.6 (5.7)	24	42.8 (4.8)	41.7 (5.4)	0.59
Trunk fat (%)	13	43 (6.1)	24	43.8 (5.3)	42.6 (5.4)	0.84
Subcutaneous adipose tissue (L)	13	7.7 (2.5)	24	8.5 (3.7)	7 (2.2)	0.53
Visceral adipose tissue (L)	13	1.8 (0.7)	24	1.8 (0.7)	2.2 (1)	0.29
Liver fat content (%)	13	6.8 (5.2)	24	8.8 (5.8)	17.9 (11.3)	**<0.001**
Liver stiffness (kPa)	13	2.3 (0.2)	24	2.4 (0.3)	2.7 (0.5)	**0.01**
Liver fibrosis (N, %)	13	1 (7.7)	24	2 (8.3)	7 (31.8)	0.06
Dietary Intake						
Energy intake (kcal/day)	13	1856 (779)	24	1479 (384)	1639 (607)	0.28
Fat (g/day)	13	68 (30)	24	55 (17)	57 (28)	0.20
Protein (g/day)	13	69 (28)	24	63 (20)	72 (28)	0.72
Carbohydrates (g/day)	13	248 (113)	24	186 (69)	214 (82)	0.25
Total sugars (g/day)	13	86 (38)	24	80 (39)	94 (49)	0.57
Added sugars (g/day)	13	54 (39)	24	44 (28)	45 (39)	0.49
Sucrose(g/day)	13	30 (17)	24	36 (27)	35 (26)	0.57
Fiber (g/day)	13	18 (8)	24	12 (5)	16 (5)	0.22
Dietary fatty acids profile						
Saturated fatty acids (g/day)	13	22 (9)		19 (7)	18 (9)	0.20
MUFA (g/day)	13	23 (10)		19 (7)	19 (9)	0.12
PUFA (g/day)	13	18 (12)		13 (5)	13 (8)	0.13
Omega-3 fatty acids (g/day)	13	1.8 (1.4)		1.2 (0.4)	1.4 (0.9)	0.25
EPA (g/day)	13	0.01 (0.03)		0.02 (0.04)	0.01 (0.01)	0.84
DHA (g/day)	13	0.02 (0.03)		0.05 (0.08)	0.03 (0.04)	0.55
Omega-6 fatty acids (g/day)	13	15.9 (10.5)		11.7 (4.8)	11.9 (7.1)	0.12
Arachidonic acid (g/day)	13	0.1 (0.07)		0.1 (0.07)	0.15 (0.1)	0.08
Linoleic acid (g/day)	13	15.8 (10.4)		11.5 (4.8)	11.7 (7.0)	0.11
Saturated fatty acids (g/day)	13	22 (9)	24	19 (7)	18 (9)	0.20

CC, *PNPLA3* genotype CC; CG, *PNPLA3* genotype CG; GG, *PNPLA3* genotype GG; MUFA, monounsaturated fatty acids; PUFA, polyunsaturated fatty acids; EPA, Eicosapentaenoic fatty acid; DHA, Docosahexaenoic fatty acid. *p*-values in bold represent significant difference across genotypes.

**Table 2:** Associations of biological characteristics with liver fibrosis in Hispanic adolescents with obesity. Linear models adjusted for genotype and sex.

	β	*p*-Value
Age (year)	−0.03	0.18
Sex *	-	0.008
PNPLA Genotype (GG vs. CG/CC) *	-	0.004
BMI (kg/m^2^)	−0.0003	0.97
Waist circumference (cm)	0.003	0.44
Body fat percent (%)	−0.004	0.64
Trunk fat percent (%)	−0.004	0.69
Subcutaneous adipose tissue (L)	−0.009	0.59
Visceral adipose tissue (L)	0.04	0.47
Liver fat (%)	0.01	0.05

* The association between categorical variables (sex and genotype) and liver fibrosis were performed with univariate linear models.

## Data Availability

All data used for this study can be found via the following link: https://github.com/rbarner/liverFibrosisPredictors (accessed on 10 May 2021).

## Supplementary Materials

The following are available online at https://www.mdpi.com/article/10.3390/nu13051621/s1, Figure S1. Association between dietary arachidonic acid intake and meat consumption of participants in the study, Figure S2. Association between dietary linoleic acid and oleic acid intakes and total plant fats, condiments and sauces consumption of participants in the study, Table S1. Liver fibrosis as a function of sex and PNPLA3 genotype, Table S2. Influence of dietary components on liver fibrosis in Hispanic adolescents with obesity and differential associations by PNPLA3 genotype.

## References

[1] Yang M., Gong S., Ye S.Q., Lyman B., Geng L., Chen P., Li D. (2014). Non-Alcoholic Fatty Liver Disease in Children: Focus on Nutritional Interventions. Nutrients.

[2] Anderson E.L., Howe L.D., Jones H.E., Higgins J.P.T., Lawlor D.A., Fraser A. (2015). The Prevalence of Non-Alcoholic Fatty Liver Disease in Children and Adolescents: A Systematic Review and Meta-Analysis. PLoS ONE.

[3] Welsh J.A., Karpen S., Vos M.B. (2013). Increasing prevalence of nonalcoholic fatty liver disease among United States adolescents, 1988–1994 to 2007–2010. J. Pediatrics.

[4] Than N.N., Newsome P.N. (2015). A concise review of non-alcoholic fatty liver disease. Atherosclerosis.

[5] Vos M.B., Abrams S.H., Barlow S.E., Caprio S., Daniels S.R., Kohli R., Mouzaki M., Sathya P., Jeffrey B., Sundaram S.S. (2017). NASPGHAN Clinical Practice Guideline for the Diagnosis and Treatment of Nonalcoholic Fatty Liver Disease in Children: Recommendations from the Expert Committee on NAFLD (ECON) and the North American Society of Pediatric Gastroenterology, Hepatology and Nu. J. Pediatric Gastroenterol. Nutr..

[6] Ekstedt M., Hagström H., Nasr P., Fredrikson M. (2015). Fibrosis stage is the strongest predictor for disease-specific mortality in NAFLD after up to 33 years of follow-up. Hepatology.

[7] Yki-järvinen H. (2014). Non-alcoholic fatty liver disease as a cause and a consequence of metabolic syndrome. Lancet Diabetes Endocrinol..

[8] Cook L.T., O’Reilly G.A., Goran M.I., Weigensberg M.J., Spruijt-Metz D., Davis J.N. (2014). Vegetable Consumption Is Linked to Decreased Visceral and Liver Fat and Improved Insulin Resistance in Overweight Latino Youth. J. Acad. Nutr. Diet..

[9] Park S.H., Jeon W.O.O.K., Kim S.H., Kim H.J., Park D.I. (2006). Prevalence and risk factors of non-alcoholic fatty liver disease among Korean adults. J. Gastroenterol. Hepatol..

[10] Romeo S., Kozlitina J., Xing C., Pertsemlidis A., Cox D., Pennacchio L.A., Boerwinkle E., Cohen J.C., Hobbs H.H. (2008). Genetic variation in PNPLA3 confers susceptibility to nonalcoholic fatty liver disease. Nat. Genet..

[11] Browning J.D., Szczepaniak L.S., Dobbins R., Nuremberg P., Horton J.D., Cohen J.C., Grundy S.M., Hobbs H.H. (2004). Prevalence of hepatic steatosis in an urban population in the United States: Impact of ethnicity. Hepatology.

[12] Martínez L.A., Larrieta E., Calva J.J., Kershenobich D., Torre A. (2019). The Expression of PNPLA3 Polymorphism could be the Key for Severe Liver Disease in NAFLD in Hispanic Population. Ann. Hepatol..

[13] Nelson J.R., Raskin S. (2019). The eicosapentaenoic acid: Arachidonic acid ratio and its clinical utility in cardiovascular disease. Postgrad. Med..

[14] Simopoulos A.P. (2008). The Importance of the Omega-6/Omega-3 Fatty Acid Ratio in Cardiovascular Disease and Other Chronic Diseases. Exp. Biol. Med..

[15] Tomita K., Teratani T., Suzuki T., Shimizu M., Sato H., Narimatsu K., Okada Y., Kurihara C., Irie R., Yokoyama H. (2014). Free Cholesterol Accumulation in Hepatic Stellate Cells: Mechanism of Liver Fibrosis Aggravation in Nonalcoholic Steatohepatitis in Mice. Hepatology.

[16] Sun M., Kisseleva T., Diego S., Jolla L. (2015). Reversibility of liver fibrosis. Clin. Res. Hepatol. Gastroenterol..

[17] Bataller R., Brenner D.A. (2005). Liver fibrosis. J. Clin. Investig..

[18] Davis J.N., Lê K.-A., Walker R.W., Vikman S., Spruijt-Metz D., Weigensberg M.J., Allayee H., Goran M.I. (2010). Increased hepatic fat in overweight Hispanic youth influenced by interaction between genetic variation in PNPLA3 and high dietary carbohydrate and sugar consumption. Am. J. Clin. Nutr..

[19] Jones R.B., Alderete T.L., Martin A.A., Geary B.A., Hwang D.H., Palmer S.L., Goran M.I. (2018). Probiotic supplementation increases obesity with no detectable effects on liver fat or gut microbiota in obese Hispanic adolescents: A 16-week, randomized, placebo-controlled trial. Pediatric Obes..

[20] Sawh M.C., Newton K.P., Goyal N.P., Angeles J.E., Harlow K., Bross C. (2019). Normal Range for MR Elastography Measured Liver Stiffness in Children Without Liver Disease. J. Magn. Reson Imaging.

[21] Shen J., Wong G.L.-H., Chan H.L.-Y., Chan H.-Y., Yeung D.K.-W., Chan R.S.-M., Chim A.M.-L., Chan A.W.-H., Choi P.C.-L., Woo J. (2014). PNPLA3 gene polymorphism accounts for fatty liver in community subjects without metabolic syndrome. Aliment. Pharm..

[22] Goran M.I., Walker R., Le K., Mahurkar S., Vikman S., Davis J.N., Spruijt-metz D., Weigensberg M.J., Allayee H. (2010). Effects of PNPLA3 on Liver Fat and Metabolic Profile in Hispanic Children and Adolescents. Diabetes.

[23] Wagenknecht L.E., Palmer N.D., Bowden D.W., Rotter J.I., Norris M., Ziegler J., Chen Y.I., Haffner S., Scherzinger A., Carl D. (2011). NIH Public Access. Liver Int..

[24] Li Q., Qu H., Rentfro A.R., Grove M.L., Lu Y., Hanis C.L., Fallon M.B., Boerwinkle E., Fisher-hoch S.P., Mccormick J.B. (2012). NIH Public Access. Clin. Investig. Med..

[25] Valenti L., Al-serri A., Daly A.K., Galmozzi E., Rametta R., Dongiovanni P., Nobili V., Mozzi E., Roviaro G., Vanni E. (2010). Homozygosity for the Patatin-Like Phospholipase-3/Adiponutrin I148M Polymorphism Influences Liver Fibrosis in Patients with Nonalcoholic Fatty Liver Disease. Hepatology.

[26] Allemand D., Reinehr T., Widhalm K., Holl R.W. (2010). Obese boys at increased risk for nonalcoholic liver disease: Evaluation of 16 390 overweight or obese children and adolescents. Int. J. Obes..

[27] Ballestri S., Nascimbeni F., Baldelli E., Marrazzo A., Romagnoli D., Lonardo A. (2017). NAFLD as a Sexual Dimorphic Disease: Role of Gender and Reproductive Status in the Development and Progression of Nonalcoholic Fatty Liver Disease and Inherent Cardiovascular Risk. Adv. Ther..

[28] Tricò D., Caprio S., Umano G.R., Pierpont B., Nouws J., Galderisi A., Kim G., Mata M.M., Santoro N. (2018). Metabolic Features of Nonalcoholic Fatty Liver (NAFL) in Obese Adolescents: Findings From a Multiethnic Cohort. Hepatology.

[29] Sonnweber T., Pizzini A., Nairz M., Weiss G., Tancevski I. (2018). Arachidonic Acid Metabolites in Cardiovascular and Metabolic Diseases. Int. J. Mol. Sci..

[30] Burns J.L., Nakamura M.T., Ma D.W.L. (2018). Differentiating the biological effects of linoleic acid from arachidonic acid in health and disease. Prostaglandins Leukot. Essent. Fat. Acids.

[31] Innes J.K., Calder P.C. (2018). Omega-6 fatty acids and inflammation. Prostaglandins Leukot. Essent. Fat. Acids.

[32] Scorletti E., Byrne C.D. (2013). Omega-3 Fatty Acids, Hepatic Lipid Metabolism, and Nonalcoholic Fatty Liver Disease. Annu. Rev. Nutr..

[33] López-Vicario C., González-Périz A., Rius B., Morán-Salvador E., García-Alonso V., Lozano J.J., Bataller R., Kang J.X., Arroyo V., Clària J. (2014). Molecular interplay between Δ5/Δ6 desaturases and long-chain fatty acids in the pathogenesis of non-alcoholic steatohepatitis. Gut.

[34] Araya J., Rodrigo R., Pettinelli P., Araya A.V., Poniachik J., Videla L.A. (2010). Decreased Liver Fatty Acid Δ-6 and Δ-5 Desaturase Activity in Obese Patients. Obesity.

[35] Adam O., Beringer C., Kless T., Lemmen C., Adam A., Wiseman M., Adam P., Klimmek R., Forth W. (2003). Anti-inflammatory effects of a low arachidonic acid diet and fish oil in patients with rheumatoid arthritis. Rheumatol. Int..

[36] Ariyoshi K., Okuya S., Kunitsugu I., Matsunaga K., Nagao Y., Nomiyama R., Takeda K., Tanizawa Y. (2015). Ultrasound analysis of gray-scale median value of carotid plaques is a useful reference index for cerebro-cardiovascular events in patients with type 2 diabetes. J. Diabetes Investig..

[37] Ishitobi T., Hyogo H., Kan H., Hiramatsu A., Arihiro K., Aikata H., Chayama K. (2015). Eicosapentaenoic acid/arachidonic acid ratio as a possible link between non-alcoholic fatty liver disease and cardiovascular disease. Hepatol. Res..

[38] Molendi-coste O., Legry V., Leclercq I.A. (2011). Why and How Meet n-3 PUFA Dietary Recommendations?. Gastroenterol. Res. Pract..

[39] Hodson L., Bhatia L., Scorletti E., Smith D.E., Jackson N.C., Umpleby M., Calder P.C., Byrne C.D. (2017). Docosahexaenoic acid enrichment in NAFLD is associated with improvements in hepatic metabolism and hepatic insulin sensitivity: A pilot study This article has been corrected since Advance Online Publication and a corrigendum is also printed in this issue. Eur. J. Clin. Nutr..

[40] Yan J., Guan B., Gao H., Peng X. (2018). Omega-3 polyunsaturated fatty acid supplementation and non-alcoholic fatty liver disease. Med. Baltim..

[41] Janczyk W., Lebensztejn D., Wierzbicka-ruci A. (2015). Omega-3 Fatty Acids Therapy in Children with Nonalcoholic Fatty Liver Disease: A Randomized Controlled Trial. J. Pediatrics.

[42] Scorletti E., Bhatia L., Mccormick K.G., Clough G.F., Nash K., Hodson L., Moyses H.E., Calder P.C., Byrne C.D. (2014). Effects of Purified Eicosapentaenoic and Docosahexaenoic Acids in Nonalcoholic Fatty Liver Disease: Results From the WELCOME* Study. Hepatology.

[43] Romeo S., Huang-Doran I., Baroni M.G., Kotronen A. (2010). Unravelling the pathogenesis of fatty liver disease: Patatin-like phospholipase domain-containing 3 protein. Curr. Opin. Lipidol..

[44] He S., McPhaul C., Li J.Z., Garuti R., Kinch L., Grishin N.V., Cohen J.C., Hobbs H.H. (2010). A Sequence Variation (I148M) in PNPLA3 Associated with Nonalcoholic Fatty Liver Disease Disrupts Triglyceride Hydrolysis. J. Biol. Chem..

[45] Anstee Q.M., Day C.P. (2015). The Genetics of Nonalcoholic Fatty Liver Disease: Spotlight on PNPLA3 and TM6SF2. Semin. Liver Dis..

[46] Huang Y., He S., Li J.Z., Seo Y.-K., Osborne T.F., Cohen J.C., Hobbs H.H. (2010). A feed-forward loop amplifies nutritional regulation of PNPLA3. Proc. Natl. Acad. Sci. USA.

[47] Li J.Z., Huang Y., Karaman R., Ivanova P.T., Brown H.A., Roddy T., Castro-Perez J., Cohen J.C., Hobbs H.H. (2012). Chronic overexpression of PNPLA3I148M in mouse liver causes hepatic steatosis. J. Clin. Investig..

[48] Nanji A.A., French S.W. (1989). Dietary linoleic acid is required for development of experimentally induced alcoholic liver injury. Life Sci..

[49] Jeyapal S., Kona S.R., Mullapudi S.V., Putcha U.K., Gurumurthy P., Ibrahim A. (2018). Substitution of linoleic acid with α-linolenic acid or long chain n-3 polyunsaturated fatty acid prevents Western diet induced nonalcoholic steatohepatitis. Sci. Rep..

[50] Okamoto Y., Tanaka S., Haga Y. (2002). Enhanced GLUT2 gene expression in an oleic acid-induced in vitro fatty liver model. Hepatol. Res..

[51] Janorkar A.V., King K.R., Megeed Z., Yarmush M.L. (2009). Development of an in vitro cell culture model of hepatic steatosis using hepatocyte-derived reporter cells. Biotechnol. Bioeng..

[52] Cansanção K., Silva Monteiro L., Carvalho Leite N., Dávalos A., Tavares do Carmo M.D.G., Arantes Ferreira Peres W. (2018). Advanced Liver Fibrosis Is Independently Associated with Palmitic Acid and Insulin Levels in Patients with Non-Alcoholic Fatty Liver Disease. Nutrients.

