# Supplementary material for: PNPLA3 Genotype, Arachidonic Acid Intake, and Unsaturated Fat Intake Influences Liver Fibrosis in Hispanic Youth with Obesity
Source: Nutrients. 2021 May 12;13(5):1621. doi: 10.3390/nu13051621 (PMC8151324; doi:10.3390/nu13051621)
Supplement: Supplementary file 1 [file nutrients-13-01621-s001.zip › nutrients-1190158-supplementary.pdf]

Table S1. Liver fibrosis as a function of sex and *PNPLA3* genotype

	**Model 1**		**Model 2**		**Model 3**		**Model 4**	
	Effect Size (η^2*100)	*p*-value	Effect Size (η^2*100)	*p*-value	Effect Size (η^2*100)	*p*-value	Effect Size (η^2*100)	*p*-value
Sex	8.39	**0.02**	7.64	**0.022**	1.26	0.339	1.40	0.316
Genotype	10.14	**0.008**	8.04	**0.019**	0.58	0.515	0.72	0.471
Sex*Genotype Interaction					3.77	0.101	3.95	0.096

Model 1: with no adjustments;
Model 2: adjusted for liver fat;
Model 3: includes the interaction between sex and *PNPLA3* genotype with no adjustments;
Model 4: includes the interaction between sex and *PNPLA3* genotype adjusted for liver fat

Table S2. Influence of dietary components on liver fibrosis in Hispanic adolescents with obesity and differential associations by *PNPLA3* genotype.

	**Dietary influence on liver fibrosis***		**Dietary influence on liver fibrosis by *PNPLA3* genotype***		
	β	***p*-value**	β_{GG}	$\beta_{CC/CG}$	**Interaction *p*-value**
**Energy intake (kcal/day)**	$2.66x10^{-6}$	0.96	$1.86\ x10^{-4}$	$-1.22\ x10^{-4}$	**0.007**
**Fat (g/day)**	$3.88x10^{-3}$	0.22	$6.78\ x10^{-3}$	$1.88\ x10^{-4}$	**0.01**
**Protein (g/day)**	$1.83x10^{-3}$	0.35	$4.59\ x10^{-3}$	$-1.57\ x10^{-3}$	0.02
**Carbohydrates (g/day)**	$-1.53x10^{-3}$	0.15	$-1.06\ x10^{-4}$	$-1.74\ x10^{-3}$	0.048
**Total sugars (g/day)**	$-2.36x10^{-4}$	0.86	$1.32\ x10^{-3}$	$-1.68\ x10^{-3}$	0.06
**Added sugars (g/day)**	$-1.44x10^{-3}$	0.39	$4.52\ x10^{-4}$	$-3.18\ x10^{-3}$	0.06
**Sucrose(g/day)**	$-2.62x10^{-4}$	0.89	$3.26\ x10^{-3}$	$-1.97\ x10^{-3}$	0.09
**Fiber (g/day)**	$-6.8x10^{-4}$	0.92	$1.14\ x10^{-2}$	$-5.18\ x10^{-3}$	0.21
	**Dietary Fatty Acids profile**				
**Saturated fatty acids (g/day)**	$4.18x10^{-3}$	0.46	$7.11\ x10^{-3}$	$-1.43\ x10^{-3}$	0.26
**Unsaturated fatty acids (g/day)**	$8.86x10^{-3}$	0.08	$1.5\ x10^{-2}$	$2.91\ x10^{-3}$	0.02
**MUFA (g/day)**	$1.01x10^{-2}$	0.17	$2.12\ x10^{-2}$	$1.52\ x10^{-3}$	**0.01**
**Oleic acid (g/day)**	$1.01\ x10^{-2}$	0.20	$2.24\ x10^{-2}$	$1.26\ x10^{-3}$	**0.01**
**PUFA (g/day)**	$1.04\ x10^{-2}$	0.20	$2.13\ x10^{-2}$	$-4.12\ x10^{-3}$	**0.01**
**Omega-3 fatty acids (g/day)**	$4.75\ x10^{-2}$	0.52	$1.31\ x10^{-1}$	$-9.92\ x10^{-2}$	0.02
**EPA (g/day)**	-1.19	0.54	21.2	-3.34	**0.0002**
**DHA (g/day)**	0.61	0.34	4.19	$-2.18\ x10^{-2}$	0.02
**Omega-6 fatty acids (g/day)**	$1.16x10^{-2}$	0.19	$2.48\ x10^{-2}$	$-3.73\ x10^{-3}$	**0.01**
**Arachidonic acid (g/day)**	0.95	**0.03**	1.87	-0.22	0.02
**Linoleic acid (g/day)**	$1.14x10^{-2}$	0.20	$2.49\ x10^{-2}$	$-3.79\ x10^{-3}$	**0.01**

*Models adjust for age, gender and energy intake

MUFA, monounsaturated fatty acids; PUFA, polyunsaturated fatty acids; EPA, Eicosapentaenoic fatty acid; DHA, Docosahexaenoic fatty acid

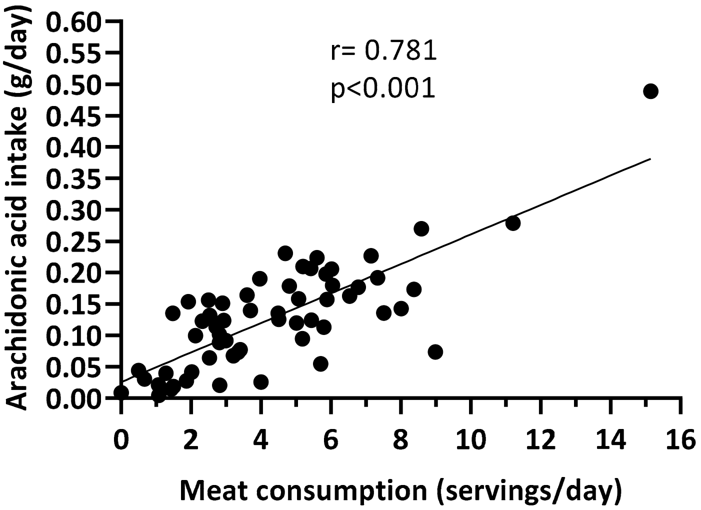

Figure S1. Association between dietary arachidonic acid intake and meat consumption of participants in the study. A linear model was used to determine the association between arachidonic acid intake (g/day) and meat consumption (servings/day). This model additionally adjusted for caloric intake.

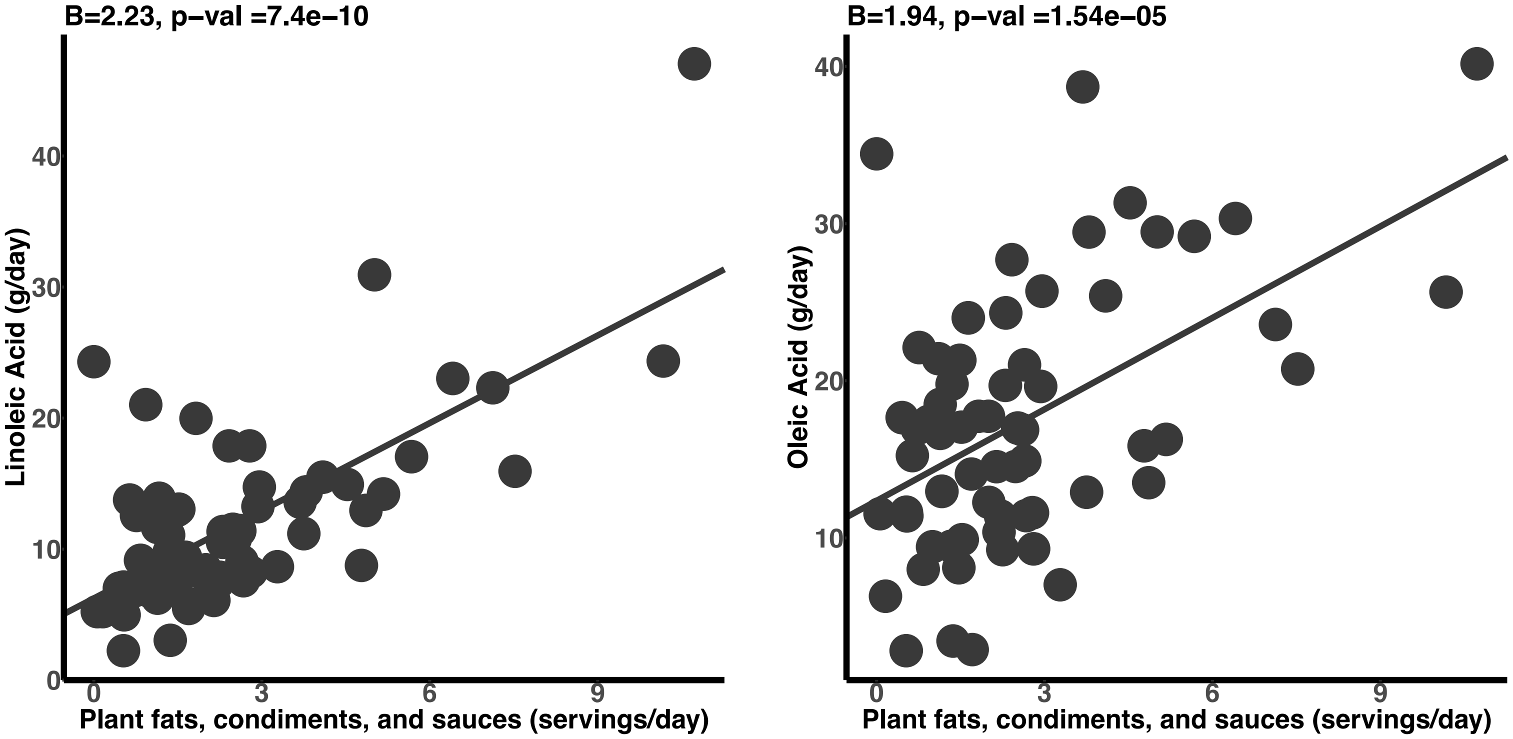

(a) **(b)**

Figure S2. Association between dietary linoleic acid and oleic acid intakes and total plant fats, condiments and sauces consumption of participants in the study. A linear model was used to determine the association between (a) linoleic acid intake (g/day) or (b) oleic acid intake (g/day) and total plant fats, condiments and sauces consumption (servings/day).
